# Sensorimotor Coarticulation in the Execution and Recognition of Intentional Actions

**DOI:** 10.3389/fpsyg.2017.00237

**Published:** 2017-02-23

**Authors:** Francesco Donnarumma, Haris Dindo, Giovanni Pezzulo

**Affiliations:** ^1^Institute of Cognitive Sciences and Technologies, National Research CouncilRome, Italy; ^2^Computer Science Engineering, University of PalermoPalermo, Italy

**Keywords:** coarticulation, joint action, action recognition, planning, distal actions, sequential action

## Abstract

Humans excel at recognizing (or inferring) another's distal intentions, and recent experiments suggest that this may be possible using only subtle kinematic cues elicited during early phases of movement. Still, the cognitive and computational mechanisms underlying the recognition of intentional (sequential) actions are incompletely known and it is unclear whether kinematic cues alone are sufficient for this task, or if it instead requires additional mechanisms (e.g., prior information) that may be more difficult to fully characterize in empirical studies. Here we present a computationally-guided analysis of the execution and recognition of intentional actions that is rooted in theories of motor control and the coarticulation of sequential actions. In our simulations, when a performer agent coarticulates two successive actions in an action sequence (e.g., “reach-to-grasp” a bottle and “grasp-to-pour”), he automatically produces kinematic cues that an observer agent can reliably use to recognize the performer's intention early on, during the execution of the first part of the sequence. This analysis lends computational-level support for the idea that kinematic cues may be sufficiently informative for early intention recognition. Furthermore, it suggests that the social benefits of coarticulation may be a byproduct of a fundamental imperative to optimize sequential actions. Finally, we discuss possible ways a performer agent may combine automatic (coarticulation) and strategic (signaling) ways to facilitate, or hinder, an observer's action recognition processes.

## 1. Introduction

Imagine a football player who is approaching the opponent team's area with the ball. One can define the player's current goal as approaching the area, while his distal intention is passing the ball or shooting. For both his teammates and his opponents, inferring the player's distal intention (not only his current goal) offers an advance opportunity to help or hinder him, highlighting the importance of goal and intention recognition in realistic social interactions, cooperative or competitive. From a computational perspective, another's proximal goals and distal intentions can be considered *hidden* (i.e., non-observable) cognitive variables that can be inferred based on observables (e.g., the player's behavior) and prior knowledge (e.g., tactics used by the soccer team) (Wolpert et al., 2003). While generally difficult in real-world social settings, goal and intention recognition may be less formidable than commonly believed, because proximal kinematics turn out to be very informative.

A series of studies have shown that humans are surprisingly good at inferring another person's proximal goals or distal intentions, even with apparently little data (Sartori et al., 2009). One recent study reveals that participants who observed grasping movements were able to report accurately whether the to-be-grasped object was small or big as early as 80 ms after movement onset, suggesting that action kinematics can be very informative at early perceptual stages (Ansuini et al., 2016). A similar case may be made for the recognition of distal intentions. For example, considering the case in which an agent makes a decision between “grasping a bottle to pour water” vs. “to move the bottle,” evidence shows that the agent's decision is already discriminable by the first part of the motor action, i.e., the grasping of the bottle (Sartori et al., 2011). In fact, the way in which the bottle is grasped turns out to be slightly different (e.g., at the level of action kinematics) in the two cases. More in general, many studies show a “tendency to grasp objects differently depending on what one plans to do with the objects” (Rosenbaum et al., 2012), which means that hand preshape can be used as a cue to infer the distal intention. This situation has equivalents in other domains, such as for example linguistics, in which it is widely known that the pronunciation of segments depends on other segments which are close to them, e.g., the next segment (*coarticulation*, see e.g., Fowler, 1980; Fowler and Saltzman, 1993; Mahr et al., 2015). These subtle changes in the action kinematics provide information about the performer's goals (Sartori et al., 2009; Neal and Kilner, 2010; Manera et al., 2011; Becchio et al., 2012; Naish et al., 2013; Quesque et al., 2013; Ansuini et al., 2015; Lewkowicz et al., 2015; Cavallo et al., 2016). At least in some cases, even subtle cues are detectable and can help observers to infer the performer's distal intentions early on, thus resulting in communicative and not only pragmatic effects.

The informativeness of early kinematic cues may even increase in explicitly cooperative social settings. For example, one study reveals that during the same motor action of placing an object, the deceleration phase was found to be slower when a “giving” action (proximal goal) was directed to another individual than when it was performed without this social constrain (Becchio et al., 2008). A series of other studies have shown that, when engaged in social interactions, co-actors usually *signal* their intentions and carve their action kinematics in ways that make their action goals easier to discriminate, when there is asymmetric information and the performer agent is more knowledgeable than the observer about the task at hand (Vesper et al., 2010; Pezzulo, 2011; Pezzulo and Dindo, 2013; Pezzulo et al., 2013a; Sacheli et al., 2013; Candidi et al., 2015).

These and other studies have assessed the usefulness of (early) kinematic cues for understanding an actor's proximal goals but also his distal intentions. One possible explanation for this phenomenon is that, in the context of grasping actions, an object can be handled and manipulated differently depending on a performer's intention—hence the agent's intention can be inferred from the way the agent performs the motor action. This explanation, however, lacks a quantitative (or computational) characterization so far and it is unclear whether one can derive the benefits of distal intention recognition from normative principles, e.g., the minimization of action costs. Furthermore, it is unclear if the explanation is *sufficient* to explain the data; for example, if appealing to early kinematic cues can fully explain the rapidity of intention recognition found in human studies, or if it is instead necessary to appeal to additional mechanisms (e.g., sophisticated prior information or evolutionary adaptations for intention recognition that are fundamentally different from those that permit recognizing proximal action goals).

In this paper, we offer a computationally-guided explanation of distal intention recognition that is rooted in normative theories of computational motor control and (embodied) sequential action (Sandamirskaya and Schöner, 2010; Rosenbaum et al., 2012; Pezzulo et al., 2014, 2017; Lepora and Pezzulo, 2015; Pezzulo and Cisek, 2016). In a control theoretic perspective, proximal actions have to simultaneously fulfill the concurrent demands of proximal and distal goals (or first-order and higher-order planning). In other words, any goal-directed action is shaped according to its proximal and distal goals: *first-order planning* (associated with proximal goals) determines object handling grasp trajectory according to immediate task demands (e.g., tuning to the orientation or the grip size for an object to be grasped); *higher-order planning* (associated with distal goals) alters one's object manipulation behavior not only on the basis of immediate task demands but also on the basis of the next tasks to be performed. This would imply that in certain conditions one can impose a cost on the proximal action or execute it suboptimally in order to fulfill the requirements of a distal action, e.g., a waiter can grasp a glass with a thumb-down posture if he has to successively turn it upright (Rosenbaum et al., 1990). The necessity of simultaneously optimizing proximal and distal components of an action sequence (e.g., “reaching and grasping a bottle to pour water” vs. “to move the bottle”) implies the coarticulation of consecutive motor acts, which would thus provide a normative rationale for the differences in the former part of the sequence (“reaching and grasping the bottle”) depending on the latter part or distal intention^1^.

Below we present a computational analysis of coarticulation during object grasping showing that (i) an agent who coarticulates proximal and distal actions produces different kinematic patterns in the first part of a sequential action (“reaching and grasping the bottle”) depending on his distal goal (“pouring” or “moving the bottle”); (ii) in turn, coarticulation gives rise to kinematic features that are sufficient for observers to correctly discriminate the agent's distal intention early in time—at least in some cases. Our analysis provides computational-level support for the idea that accurate intention recognition may be due to early kinematic cues elicited during proximal actions, without necessarily requiring additional mechanisms. In turn, the elicitation of informative cues may be a byproduct of the optimization of sequential actions and does not need to have necessarily a social goal (e.g., facilitation of action recognition, like in signaling Vesper et al., 2010; Pezzulo, 2011; Sacheli et al., 2013)—although of course automatic (coarticulation) and strategic (signaling) modulations of one's own action kinematics can be merged.

## 2. Computational approach

In computational motor control, it is widely assumed that action representations stem from (probabilistic) internal models (Wolpert et al., 2003; Jeannerod, 2006; Shadmehr and Krakauer, 2008; Friston et al., 2010, 2017; Pezzulo et al., 2015, in press; Donnarumma et al., 2016; Maisto et al., 2016; Stoianov et al., 2016). These models can be hierarchical, with higher hierarchical levels encoding more abstract and distal aspects and lower hierarchical levels encoding more proximal aspects that are related to action performance. At lower levels, actions such as grasping or pouring can be associated with probability distributions over hand kinematics (e.g., controls of angles of fingers), which of course change over time as the action unfolds.

Within this general probabilistic framework, we model the performer agent using a computational method that combines basic actions (or motor primitives) such as grasping and pouring to realize a sequential action (e.g., grasp a bottle to move it or pour), with or without coarticulating them. Furthermore, we model the observer agent using a computational method that infers the performer agent's current action, by “simulating” the execution of (the same) motor primitives for grasping, moving and pouring. Below we briefly introduce these two computational models, which we successively specify more formally.

### 2.1. Rationale of the computational approach

#### 2.1.1. Performer agent

**Figure 1:**
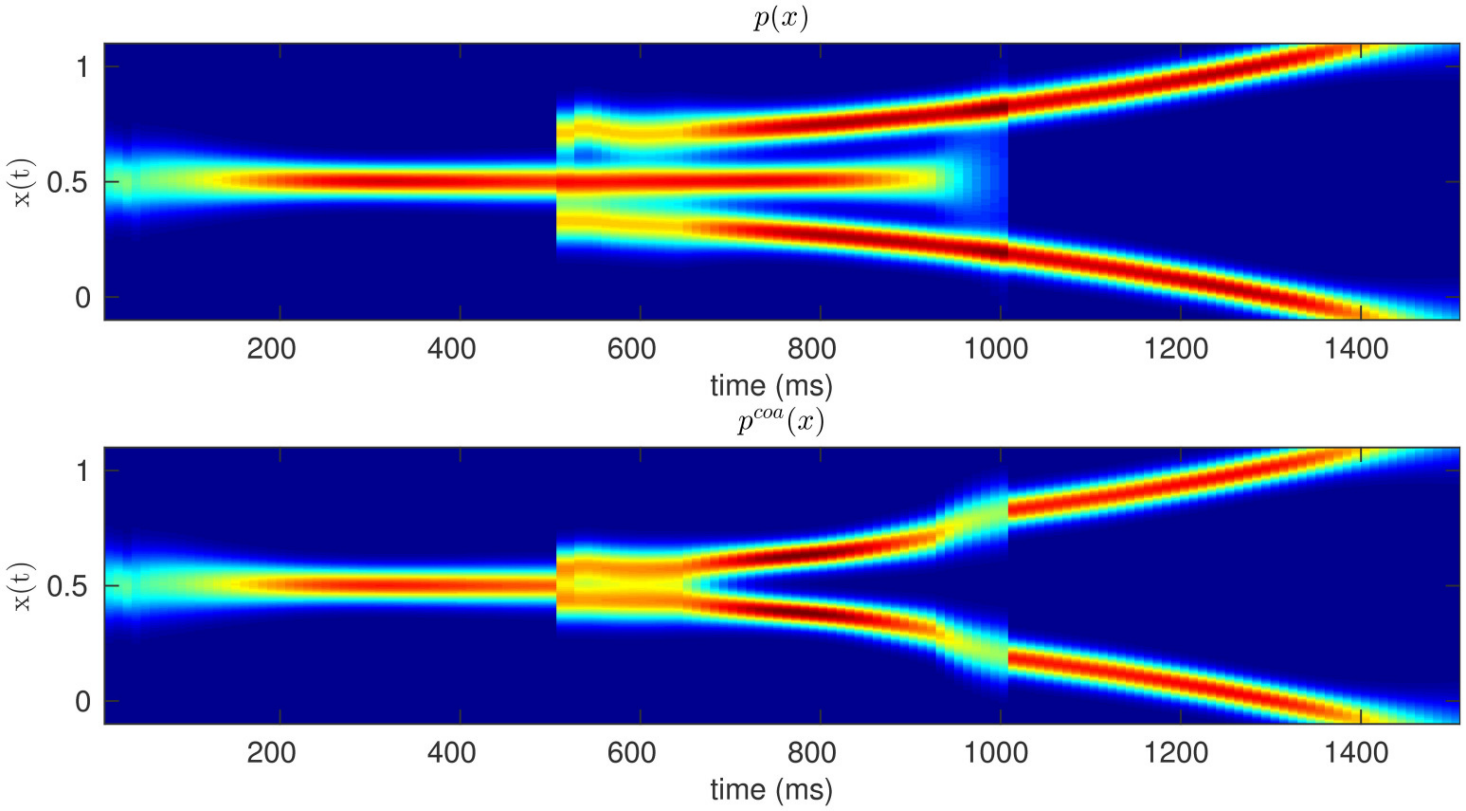
**Original (Top)** vs. Coarticulated **(Bottom)** distributions for a simplified action sequence (e.g., mouse movement). The sequence consists of two parts: moving to the center-right (from zero to 1,000 ms) and moving to the top-right or bottom-right (from 1,000 to 1,500 ms); see main text for explanation. The colors denote the probability of occupying a given position in space, during time, from red (highest probability) to blue (lowest probability).

According to our coarticulation hypothesis, we describe the planning of sequential actions as the *coarticulation* (or assimilation) of two successive motor primitives, e.g., motor primitive for reaching-and-grasping and one for grasping-and-pouring. Intuitively, assimilation implies that if the two sequential actions are modeled by two different probability distributions of hand kinematics (Dindo et al., 2011; Pezzulo et al., 2013a), these two distributions are made more similar by sampling from their probabilistic superposition (aka, coarticulated distribution) rather than the two original distributions, over time. Figure 1 offers a schematic illustration of this concept in a simplified 2D domain, where the proximal action (from time zero to time 1,000) corresponds to moving a mouse to the center-right, and the distal action (from time 1,000 to time 2,000) corresponds to moving the mouse to the top-right or bottom-right. The colors correspond to the mean and variance of the probability distributions of hypothetical center-right, top-right and bottom-right mouse movements. The figure shows how the same proximal action—move to center-right—can be either independent from (top panel) or assimilated/coarticulated with (bottom panel) the successive action of reaching the top-right or bottom-right. In the latter case, the effects of assimilation on the mouse movements are apparent from time 600, well before the (theoretical) beginning of the distal action.

#### 2.1.2. Observer agent

According to motor theories of cognition, the computational mechanisms (and internal models) used for action planning and execution are also reused for action understanding, using *motor simulation* (Jeannerod, 2006). In keeping with this idea, we model the action observation process as a (probabilistic) inference problem, in which an observer agent considers multiple possible hypotheses that correspond to the actions that may have generated the observed sensory stimuli (i.e., whether the performer agent is grasping for pouring vs. grasping for moving) and has to select one of them. To do so, the observer agent simulates executing multiple actions in parallel (from his own motor repertoire), compares the predictions under these different hypotheses with the observed movements, and assigns high probability to the action/hypothesis that generates the smaller prediction error. This process is iterated over time using a probabilistic scheme (see below), so that as the performer agent's actions unfold in time, evidence accumulates for one of the alternative hypotheses. Note that using this framework implicitly requires the assumption that performer and observer agents share the same set of motor primitives, although the probabilistic parameters might differ according to individual actor's knowledge and expertise. Our simulations will show that this motor simulation process converges more readily to the correct explanation when the performer agent uses coarticulation—and in this latter case, an observer agent can correctly recognize the distal intention of a demonstrating agent while he is still executing the proximal action.

### 2.2. Formal aspects of the computational approach

#### 2.2.1. Performer agent and the coarticulation distribution

Coarticulation is the process of altering one's own behavior to facilitate the next action. In this framework, a proximal action is coarticulated (or assimilated) with the next action in a sequence if the differences between the (probability distributions denoting the) two actions are minimized, while at the same time it maintains its correct pragmatics (e.g., a reaching action has to effectively reach the bottle despite being coarticulated with a successive grasping action).

**Figure 2:**
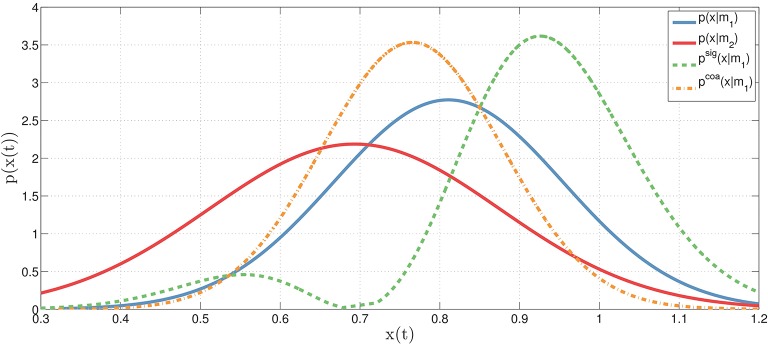
**Illustration of original, coarticulation and signaling distributions**. The original (Gaussian) distributions (at time *t*) corresponding to two motor primitives: *p*(*x*_*t*_|*m*_1_) for motor primitive *m*_1_ (blue) and *p*(*x*_*t*_|*m*_2_) for motor primitive *m*2 (red). In the *coarticulation distribution* $p^{coa}\left(x_{t}|m_{1}\right)$ (orange), the motor primitive *m*_1_ is coarticulated (or assimilated, i.e., made as similar as possible) with the motor primitive *m*_2_ using Equation (2)—where *m*_1_ and *m*_2_ may correspond, for example, to the first and second action in a sequence. In the *signaling distribution* $p^{sig}\left(x_{t}|m_{1}\right)$ (green), the motor primitive *m*_1_ is dissimilated (i.e., made as different as possible) from the motor primitive *m*_2_. See the main text for explanation.

To exemplify this concept, let's consider two actions (e.g., reaching a bottle and executing a power grasp), each implemented as a motor primitive (*m*_1_ or *m*_2_) that, for every moment in time, can be associated to a probability distribution (for example, a Gaussian distribution over its corresponding kinematic parameters such as hand and finger configurations). Figure 2 shows the distributions associated to the two motor primitives, *p*(*x*_*t*_|*m*_1_) for model *m*_1_ (e.g., reaching the bottle, in blue) and *p*(*x*_*t*_|*m*_2_) for model *m*2 (e.g., power grasp, in red), at time *t*. Based on these two *original* distributions, it is possible to compute the novel *coarticulated distribution* $p^{coa}\left(x_{t}|m_{1}\right)$ (e.g., reaching the bottle while preparing to grasp it with a power grasp, in orange), which corresponds to the fact that at time *t*, the motor primitive *m*_1_ is *coarticulated* with *m*_2_. Obviously, this example only describes what happens in a single temporal instant, while actions unfold in time. To model temporal dynamics of motor primitives, it is possible to extend the same formalism using continuous distributions, see below.

It is important to remark that any sample drawn from the coarticulation distribution (in orange) at time *t* should simultaneously satisfy two constraints: it should be representative of the original distribution of the first motor primitive *p*(**x**_*t*_|*m*_1_) while at the same time should have a high probability of belonging to the second motor primitive *m*_2_ (or in more general cases, even to a set of future motor primitives, *m*_*j*_). In keeping, to obtain an approximation of the coarticulation distribution, we adopt a *rejection sampling* technique. Let ${\hat{\text{x}}}_{t}$ be a sample from the original distribution *p*(**x**_*t*_|*m*_*i*_) or a motor primitive *m*_*i*_. Given *K* random values, *u*_*k*_∈[0, 1], sampled from the uniform distribution over $\left[0,p\left({\text{x}}_{t}|m_{k}\right)/p_{k}^{max}\right]$, we decide to *accept* the sample ${\hat{\text{x}}}_{t}$ if the following holds:

(1) $$u_{i}<w_{i}\cdot p\left({\hat{x}}_{t}|m_{i}\right)\text{ and }u_{j}<w_{j}\cdot p\left({\hat{x}}_{t}|m_{j}\right),\forall j\neq i$$

where **w** = [*w*_1_, *w*_2_, …, *w*_*K*_] is a vector of weights that modulate the contribution of the individual motor primitives in the coarticulation distribution. Intuitively, this implies that a sample is accepted if and only if it is a “good exemplar” of both (say) the grasping and the pouring distributions—implying that the novel coarticulation distribution combines aspects of grasping and pouring.

In the case of continuous distributions *p*(**x**_*t*_|*m*_*j*_), the *coarticulation* distribution becomes:

(2) $$p^{coa}\left(x_{t}|m_{i};w\right)\propto w_{i}\cdot p\left(x_{t}|m_{i}\right)\prod_{j\neq i}\left(w_{j}\cdot p\left(x_{t}|m_{j}\right)\right)$$

The resulting coarticulation distribution $p^{coa}\left(x_{t}|m_{i}\right)$ is constructed in such a way that its kinematic parameters are the most probable for the motor primitive *m*_*i*_ but also the most similar to those of the primitive(s) to be executed next (*m*_*j*_). As illustrated in Figure 1, the motor primitives for (say) grasping and pouring then mesh coherently over time (bottom panel: coarticulation), rather than being simply executed one after the other (top panel: no coarticulation). Another way to appreciate the key features of the coarticulation distribution is contrasting it with its “converse”: the *signaling* distribution, see Figure 2. While the coarticulation distribution is constructed in such a way to emphasize the similarities between two motor primitives, the signaling distribution is constructed in such a way to emphasize their differences—hence the former (coarticulation) distribution is more appropriate to model assimilation effects (e.g., between two consecutive motor primitives as in our examples) and the latter (signaling) distribution is more appropriate to model dissimilation effects such as those arising during non-verbal, sensorimotor communication (Vesper et al., 2010; Pezzulo, 2011; Pezzulo and Dindo, 2013; Pezzulo et al., 2013a,b; Sacheli et al., 2013; Candidi et al., 2015). See the Appendix for a more formal treatment of the signaling distribution.

#### 2.2.2. Observer agent and probabilistic motor simulation

**Figure 3:**
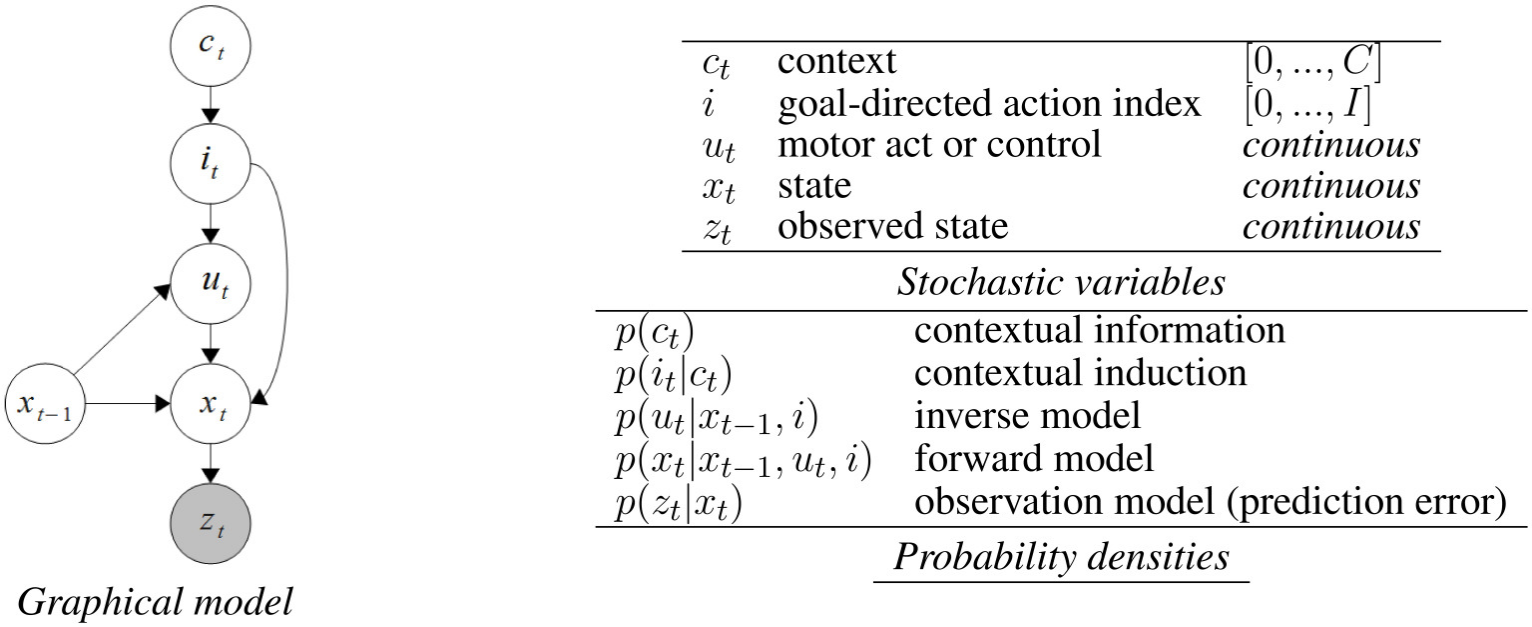
**Graphical model (DBN) for action simulation based on coupled forward-inverse models**. See main text and Dindo et al. (2011) for details.

Our implementation of action understanding via motor simulation is based on a Dynamic Bayesian Network (DBN) shown in Figure 3. DBNs are Bayesian networks representing temporal probability models in which directed arrows depict assumptions of conditional (in)dependence between variables (circles) (Murphy, 2002). Shaded nodes represent observed variables while the others are hidden and need to be estimated through the process of probabilistic inference. In our representation, the process of action understanding is influenced by the following factors expressed as stochastic variables in the model (see Dindo et al., 2011 for a more detailed account of the model):

1. *c*: discrete context variable;
2. *i*: index of the agent's own repertoire of goal-directed actions (proximal or distal): each action directly influences the activation of related forward and *inverse* models;
3. *u*: control-related continuous variable (e.g., forces, velocities, …);
4. *x*: state (e.g., the position of the demonstrator's end-effector in an allocentric reference frame);
5. *z*: observation, a perceptual measurement related to the state (e.g., the perceived position of the demonstrator's end-effector on the retina).

During action observation, the model has to infer which action the performer agent is performing (e.g., whether he or she is currently grasping, pouring, lifting, etc.—where each action, proximal or distal, is denoted by an index *i*_*t*_). The goal-directed action is considered to be hidden (i.e., not directly observable); but it can be inferred on the basis of noisy sensory observations (denoted as *z*_*t*_), e.g., the performer's hand movements. The logic is the usual of (inverse) Bayesian inference, which considers multiple potential actions as candidate explanations, which compete to explain the sensory data (Wolpert et al., 2003; Demiris and Khadhouri, 2005; Dindo et al., 2011; Friston et al., 2011; Pezzulo, 2013; Donnarumma et al., in press). Each action *i*_*t*_ is associated with a paired inverse-forward model (see Equation 4 below). Re-enacting these actions “in simulation” generates a motor control *u*_*t*_ (given the hidden state *x*_*t*−1_, aka inverse model), and a prediction of the next hidden state *x*_*t*_ (given the motor control *u*_*t*_ and the previous state *x*_*t*−1_, aka forward model). Comparing the predicted and sensed movements under various competing hypotheses (e.g., grasping, pouring) permits to assess which one generates less prediction error, hence explaining better the data. A priori contextual information *c*_*t*_ can bias the inferential process and the initial choice of the internal models to test (in case they are too numerous).

The following equations describe the observation model (Equation 3), which specifies the way (noisy) sensory stimuli are used to estimate the state of the demonstrator (e.g., hand position); the transition model (Equation 4), which specifies how the state of the demonstrator evolves as a function of his or her goals and motor commands; and the a priori distribution over the set of hidden variables (Equation 5), which represents the perceiver's prior belief and is a necessary ingredient of Bayesian systems.

(3) $$p\left({Ƶ}_{t}|X_{t}\right)=p\left(z_{t}|x_{t}\right)$$

(4) $$p\left(X_{t}|X_{t-1}\right)=p\left(x_{t}|x_{t-1},u_{t},i\right)\cdot p\left(u_{t}|x_{t-1},i\right)$$

(5) $$p\left(X_{0}\right)=p\left(x_{0}\right)\cdot pc_{0}\cdot p\left(i|c_{0}\right)$$

The inference exploits the usual (prediction) error-correction mechanisms of Bayesian systems. The model starts with prior hypotheses about the demonstrator's actions and intentions, and these are iteratively revised as new sensory evidence is sampled. The evidence provided by the perceptual process and the observed states (*z*_*t*_) is responsible for “correcting” the posterior distribution when integrating the observation model *p*(*z*_*t*_|*x*_*t*_). In other words, those parts of the hidden state that are in accordance with the observations will exhibit peaks in the posterior distribution. Since those states have been produced by a goal-directed motor primitive, the marginalization of the final posterior distribution produces the required discrete distribution over motor primitives, *p*(*i*_*t*_|*z*_1:*t*_).

Thus, the motor primitive with the highest probability (above a fixed threshold) is selected as the “winning” primitive; such an inference process can be iterated over time by using the full posterior distribution as the prior for the next step, until convergence. Ultimately, the aim of the whole process is estimating the probability of each model given the current observations so far (i.e., likelihood). The most plausible model is the one that maximizes the posterior probability of the model. As usual in a Bayesian setting, the whole process is influenced by the choice of the prior distributions for the available motor primitives: the more likely is a particular motor primitive a-priori, the more reliable and fast its recognition. In particular, using this framework action recognition is influenced by an auxiliary (contextual) variable, which can intuitively reflect an agent's contextual knowledge (e.g., that pouring is highly unlikely if the bottle is almost empty) that biases the motor primitives that are actually simulated by the agent. While prior probabilities and contextual information are important in real-life scenarios, we do not use them in our simulations.

## 3. Experimental setup and results

We performed a series of computational simulations, in which one (performer) agent executes one of two sequential actions (e.g., “reaching and grasping a bottle to pour water” vs. “reaching and grasping a bottle to move it”) in two conditions: with and without the coarticulation method explained in Section 2.2.1. At the same time, the other (observer) agent has to disambiguate these two alternatives as soon and as accurately as possible, using the probabilistic motor simulation methods introduced in Section 2.2.2. These simulations permit us to study the benefits of coarticulation, and to test the “sufficiency” hypothesis introduced earlier: namely, that kinematic features at early stages of a coarticulated action permit an observer to recognize the action. In our scenario, this means that a sequential action (e.g., “reaching and grasping a bottle to pour water”) can be discriminated already during the first (reaching) phase. Conversely, when the same action is executed without coarticulation, it can only be recognized during the second phase, after the agent has grasped the bottle.

In our simulations, for the sake of simplicity we focused on two two-step sequential actions: reach-and-pour vs. reach-and-move. In practice, we used three motor primitives: the former primitive (reach-to-grasp) corresponds to the first step in both sequences, while the other two primitives (grasp-to-pour and grasp-to-move) correspond to the two final actions to complete the first and second sequential actions, respectively. At each moment in time, from 0 ms (beginning of sequential action) to 1,500 ms (end of sequential action), each motor primitive corresponds to a probability distribution over controls of finger, thumb and wrist of a (human) hand.

The motor primitives were derived based on controls and parameters extracted from human data collected from six adult male participants. Each participant executed every primitive action 50 times, and data on angles of finger, thumb and wrist were collected using a dataglove (HumanGlove - Humanware S.r.l., Pontedera, Pisa, Italy) endowed with 16 sensors. The former (reach-to-grasp) motor primitive was acquired while participants reached an object the size of a bottle with a concave constriction (see also Sartori et al., 2011), with no knowledge of the next action to perform with it. We selected the latter two primitives (grasp-to-pour and grasp-to-move) as instances of power grasp and a precision grip actions, respectively, in which the end-position of the fingers was analogous to the positions reported by Sartori et al. (2011) while humans grasped a bottle to pour or move it, respectively.

The internal dynamical models (motor primitives) used in the simulations were obtained by regressing the aforementioned data (50 trials for 6 participants for each primitive), to obtain probability distributions over angles of finger, thumb and wrist, over time. For each motor primitive, we used an *Echo State Gaussian Process* (ESGP) (Chatzis and Demiris, 2011), a method for the Bayesian modeling of sequential data that produces a measure of confidence (or uncertainty) on the generated predictions (the model predictive density), which can be directly used within our computational approach.

In the simulations reported below, a non-coarticulated action corresponds to the first primitive (reach-to-grasp) being used for the first 1,000 ms, while one of the two remaining primitives (grasp-to-pour or grasp-to-move, depending on the task) is used for the successive 500 ms. A coarticulated action corresponds to the first primitive (reach-to-grasp) being coarticulated with one of the two remaining primitives (grasp-to-pour or grasp-to-move, depending on the task) during the interval between 500 and 1,000 ms, using the coarticulation method explained in Section 2.2.1. In other words, we derive the coarticulated actions by “meshing” two primitives, not by using separate ESGPs. Note that in the simulations, we coarticulated the index finger and thumb controls (not the wrist controls), coherent with their importance in grasping and pouring actions (Sartori et al., 2011).

**Figure 4:**
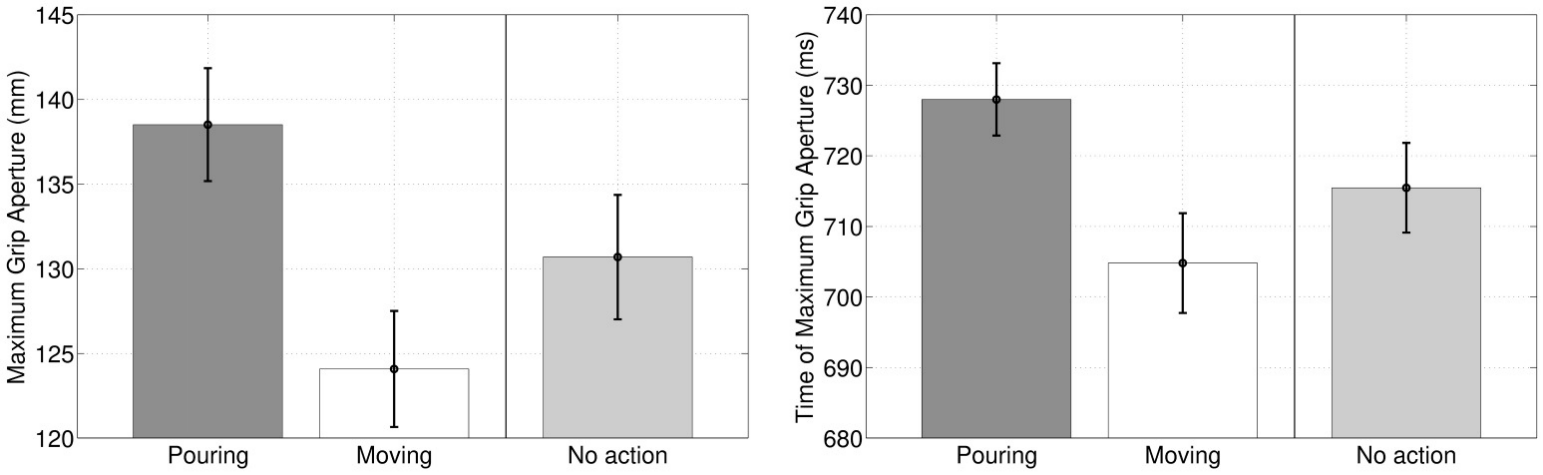
**Maximum Grip Aperture and Time of Maximum Grip Aperture when (1) the reach-to-grasp primitive is coarticulated with grasp-to-pour, (2) the reach-to-grasp primitive is coarticulated with grasp-to-move and (3) the reach-to-grasp primitive is not coarticulated (baseline condition), as if there was no successive action**.

A first result of our simulations is that during the execution of the former (reach-to-grasp) motor primitive in the sequence, Maximum Grip Aperture and Time of Maximum Grip Aperture differ significantly if the primitive is coarticulated with a grasp-to-pour primitive, with a grasp-to-move primitive, or not coarticulated at all, see Figure 4. This result is not remarkable *per se*, but can be considered as a “safety check” that the different intention elicits different action kinematics, with a pattern that is qualitatively coherent with the results of Sartori et al. (2011) in a similar scenario. What is more important for us was studying whether (and how) this difference in action kinematics translates into an advantage for the observer agent, at early stages of the performer's agent movement.

**Figure 5:**
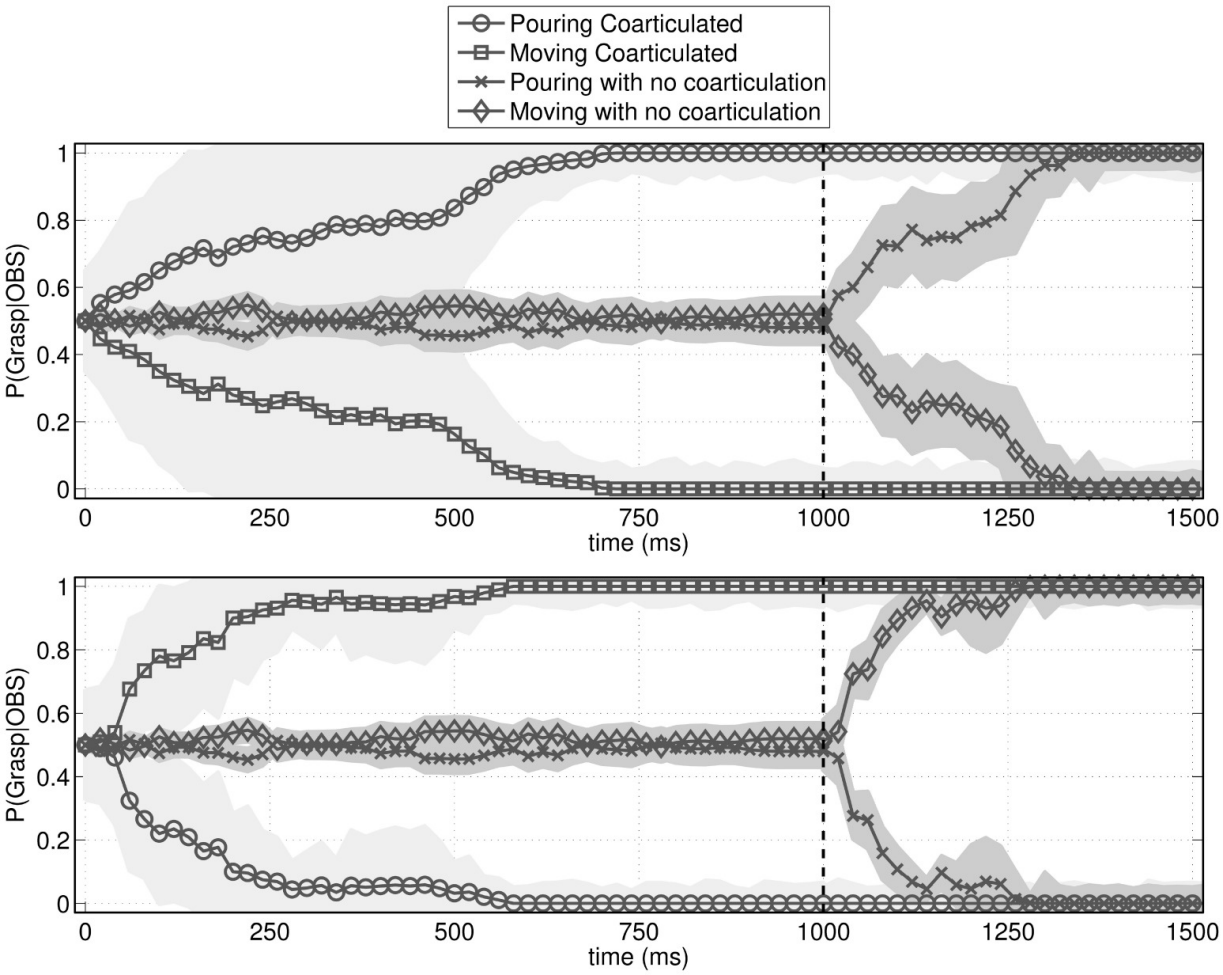
**Probability assigned by the observer agent to the to-be-recognized action (pouring or moving)**. The figures show the mean probability and standard deviation (shaded area) of pouring vs. moving action sequences given the current observations. The vertical dotted bar is the moment when the performer agent reaches the bottle. **(Top)** Probabilities over time when the to-be-recognized action is pouring. **(Bottom)** Probabilities over time when the to-be-recognized action is moving. In both cases, if the action is not coarticulated, it is recognized only after the bottle is reached. Instead, if the action is coarticulated, it is recognized early on, during the execution of the proximal action.

To answer this question, we simulated the behavior of an observer agent that has to recognize the actions performed by the performer agent, using the probabilistic motor simulation mechanism described in Section 2.2.2. As shown in Figure 5, the observer agent had a clear advantage in recognizing the performed action when it was coarticulated. More specifically, the figure shows that without coarticulation the performer agent's distal intention (pouring or moving the bottle) can be recognized only after he reaches the bottle (i.e., after time 1,000), while with coarticulation it can be recognized much earlier, during the execution of the first motor primitive (i.e., well before time 1,000). This latter result illustrates that coarticulation affords intention recognition in ways that are qualitatively different from the mere execution of a (non-informed) action.

## 4. Discussion

Humans excel at recognizing distal intentions on the basis of (apparently) little information, but the cognitive and computational mechanisms underlying this ability are incompletely known. We have proposed that normative principles regulating the coarticulation of sequential actions can explain how it is possible to infer a performer's distal intention by looking at the kinematics of his proximal actions.

To test this idea, we implemented a series of simulations in which the performer agent executes sequential actions (reach-and-pour or reach-and-move) as sequences of two primitives (reach-to-grasp and grasp-to-pour, or reach-to-grasp and grasp-to-move) with or without coarticulation. Our results show that two successive actions can be coarticulated (or assimilated) in such a way that the kinematics of the proximal action are adequate for (and informative of) the next action(s) in the sequence. Indeed, our results show that, first, coarticulated actions have characteristic kinematic features compared to non-coarticulated actions, and second, that these features may be *sufficient* for an observer agent to correctly recognize the performer's agent distal intention early on. This result holds despite the fact that we used simplified motor primitives and only coarticulated index finger and thumb controls. In principle, an observer agent having access to richer visual stimuli and more sophisticated primitives (with more controls and degrees of freedom) may enjoy additional benefits; it is however possible that coarticulation only operates on a restricted set of degrees of freedom, e.g., those that are necessary to solve the task, as for the uncontrolled manifold hypothesis (Scholz and Schöner, 1999). At the same time, it is possible that in real-life conditions, some information encoded in movement kinematics that would be potentially useful to infer a performer's intention may nevertheless remain invisible to observers—for example, when parametric variations are too small to be detected (Naish et al., 2013; Cavallo et al., 2016). Our computational study shows that coarticulation promotes the appropriate preconditions for advance intention understanding, but the additional factors that may favor (or prevent) an advantage for observer agents remain to be fully assessed.

Our emphasis on the sufficiency of kinematic cues to solve intention recognition tasks does not imply that interactive agents do not use other sources of information, such as (prior information on) the context in which the action takes place. For example, it has been argued that the same action (approaching a person with a knife) can be motivated by two different intentions (e.g., Dr. Jekyll who wants to cure or Mr. Hyde who wants to kill) and these can be disambiguated based on the place where the action occurs (operating room or dark street) (Kilner et al., 2007), but see Jacob and Jeannerod (2005); Kilner et al. (2007); Becchio et al. (2008) for alternative proposals. This kind of prior information can be readily incorporated in the action recognition scheme described above, through the contextual (*C*) node of the DBN. By considering the probabilistic relations between contexts and actions, it would be possible to bias the action recognition process and distinguish the intentions motivating two actions, even when they are kinematically identical—a situation that, as we have discussed, may be more the exception than the rule. Furthermore, it would be possible to extend the model discussed here so that it also directs saccadic eye movements to the most informative parts of the demonstrator's actions, in keeping with the idea that action recognition uses an active sensing scheme (Donnarumma et al., in press). Modeling eye movements would help understanding under which conditions subtle kinematic cues that are embedded in goal-directed actions are detected by observer agents.

Following a motor cognition approach, our model implements action recognition as a (Bayesian) inferential process that uses the logic of “analysis-by-synthesis” or action simulation (Jeannerod, 2006). This is in keeping with evidence (reviewed in Grafton, 2009) that observer agents simulate the actions they observe in their brains. Alternative hypotheses point, for example, to a more ecological or enactive view of action understanding, which appeal to “direct perception” rather than (Bayesian) inference (Gibson, 1966). While this alternative perspective would differ from our implementation, the logic of our argument here may be the same—that is, that coarticulation generates information that an observer agent can use to form an advance understanding of the performer's goals (via Bayesian inference or direct perception).

It is notable that we have illustrated the model by discussing coarticulation in the domain of reaching and grasping actions, where essentially coarticulation implies the preshaping of hands before executing a grasping action (Jeannerod, 2006). However, the phenomenon of coarticulation is evident in all sequential actions, and the model presented here is (in principle) general enough to address analogous phenomena in other domains, including speech, sign language (Jerde et al., 2003) and the planning of smooth action sequences (Rosenbaum et al., 2006). It remains to be assessed by future studies whether the computational scheme presented here is empirically adequate to explain sequential action in these and other domains, or if it needs to be extended to include more sophisticated internal generative models (e.g., of hierarchical dynamics rather than only sequences of motor primitives Kiebel et al., 2008, 2009; Donnarumma et al., 2015a,b)—as well as the relative merits of alternative frameworks such as those stemming from a dynamical systems perspective (Kelso, 1995; Marsh et al., 2006, 2009).

To sum up, according to this (normative) proposal, the main goal of coarticulation is to optimize sequential actions, and the facilitatory effects for social cognition are byproducts of this process. In other words, according to this proposal, there is no need of any action recognition or mindreading adaptation in the observer, because the action recognition process is greatly facilitated by the performer—albeit often unwittingly (but see the Appendix). This process is effective because during the execution of sequential actions, there is a sort of backward influence from the latter action (and its constraints) to the former action. Thus, the former action already includes subtle but reliable kinematic cues, which can be used to infer the performer's distal goal—and we humans excel at picking up these cues.

## Author contributions

FD, HD, and GP conceived the study, collected and analyzed data, and wrote the manuscript.

## Funding

The present research is funded by the Human Frontier Science Program (HFSP), award number RGY0088/2014, by the EU's FP7 under grant agreement no FP7-ICT-270108 (Goal-Leaders).

### Conflict of interest statement

The authors declare that the research was conducted in the absence of any commercial or financial relationships that could be construed as a potential conflict of interest.

## Footnotes

^1^For the sake of simplicity, here we equate coarticulation and assimilation, see also (Jerde et al., 2003). However, there is a conceptual difference between the two: coarticulation is the underlying process (i.e., the temporal overlap between sequential actions) while assimilation is the superficial result (in terms of increasing the similarity of the last part of the first movement to the first part of the last movement).

## Appendix

### A.1. Relations between coarticulation (or assimilation) and signaling (or dissimilating) during social interactions

While we have emphasized the automaticity of coarticulation, it can also be used *strategically* in social contexts; for example, to lower (or raise) the co-actor's uncertainty about our plans—that is, to help him or her understand an actor's own distal intentions, or to feint; or even (in principle) to smoothly combine one's own actions with those of co-actors (Gonzalez et al., 2011). To illustrate how it is possible to use coarticulation strategically, we denote with *p*(**x**|*m*_*i*_) the sequence of the states associated to the motor primitive *m*_*i*_ computed using the coarticulation distribution *p*(**x**_*t*_|*m*_*i*_). If a performer agent wants to facilitate the perceiver's action recognition process, (s)he can compute the weights **w**_*i*_(*t*) so that they minimize the following equation:

(A1) $$w_{i}(t)=argmin_{w\left(t\right)}\left[KL\left[p_{i}^{coa}(w\left(t\right)),p_{i}\right]+\lambda S\left(\theta -p_{i}^{simulated}\right)\right]$$

where:

- *KL*(·, ·) is the Kullback-Leibler divergence between the coarticulation distribution with the set of weights **w** and the distribution with no coarticulation;
- λ is the amount of coarticulation in the given action;
- $p_{i}^{simulated}$ is an estimation of the perceiving agent's posterior probability correctly recognizing the model *m*_*i*_ (under the assumption that performer and perceiver share the same set of internal models);
- θ is the (experimental) threshold that the agent uses during model recognition;
- *S* is the logistic function.

The KL term considers the cost of coarticulation, where cost can be associated to biomechanical factors, effort, and other forms of costs (e.g., cognitive costs associated to planning and executing non-familiar or non-habitual movements). The λ term permits modulation of the amount of coarticulation (λ = 0 means no coarticulation). By minimizing the above quantity, the performer agent essentially disambiguates the coarticulated action from possible alternatives, thus permitting an observer agent to infer his distal intention at early stages.

This latter example shows how it is possible to use coarticulation to *signal* one's own intentions (e.g., make them “readable”), or conversely to feint another intention, analogous to other sensorimotor communication dynamics during social interactions (Vesper et al., 2010; Pezzulo, 2011; Pezzulo and Dindo, 2013; Pezzulo et al., 2013a; Sacheli et al., 2013; Candidi et al., 2015). Indeed, in our formulation coarticulation and signaling are not just similar but stem from a consistent computational approach. Indeed, the distribution defined in Equation (2) is the dual of the *signaling* distribution defined in Pezzulo et al. (2013a), and which can be used to *dissimilate* between the current action having been performed and alternative actions, with the aim to facilitate the perceiver's agent recognition of the proximal goal.

Defining a function:

(A2) $$\begin{array}{l} p^{comm}\left(x_{t}|m_{i};w\right)\propto w_{i}\cdot p\left(x_{t}|m_{i}\right)\\ \;\prod_{k\in Dissim}\left(1-w_{k}\cdot p\left(x_{t}|m_{k}\right)/p_{k}^{max}\right)\cdot \\ \;\prod_{j\in Assim}\left(w_{j}\cdot p\left(x_{t}|m_{j}\right)\right) \end{array}$$

where $p_{k}^{max}$ is the maximum value for the distribution *p*(**x**_*t*_|*m*_*k*_), *Dissim* is the set of motor models to be dissimulated and *Assim* is the set of motor models to be coarticulated.

In short, one can use the same equation to flexibly combine or interleave assimilation and dissimilation of actions, see Figure 2. As we have shown here, one can assimilate two consecutive actions, and this would correspond to coarticulation. However, one can also assimilate two simultaneous actions, and this would correspond to a feint, in that it would render the observer's action recognition process more difficult. Finally, dissimilating one's current action from the alternatives would amount to signaling (and helpful for the observer agent), while dissimilating two consecutive actions in an action sequence would amount to feinting own's own distal intention. This equation can thus be used to derive formal descriptions of various strategies to help or hinder during social interactions, which can be helpful for the (trial-by-trial, model-based) analysis of human data (Candidi et al., 2015).
